# Challenges related to the implementation of the CCMDD programme in Sekhukhune clinics

**DOI:** 10.4102/hsag.v29i0.2707

**Published:** 2024-10-25

**Authors:** Ragosebo P. Sekopa, Robert T. Netangaheni

**Affiliations:** 1 Department of Health Studies, College of Human Sciences, University of South Africa, Pretoria, South Africa

**Keywords:** challenges, central, chronic, medicines, dispensing, distribution, programme

## Abstract

**Background:**

Implementation of the central chronic medicines dispensing and distribution (CCMDD) programme in most of Sekhukhune primary health care (PHC) has been challenging. This raised questions as to reasons for the CCMDD programme and its good intentions and benefits eventually creating challenges in the Sekhukhune district PHC facilities.

**Aim:**

This study aims to describe the challenges related to the implementation of the CCMDD programme in Sekhukhune district clinics.

**Method:**

The qualitative research approach and its explorative and descriptive research designs were used in this study. Data were acquired through one-on-one semi-structured interviews and then analysed thematically.

**Results:**

Findings of the study revealed that shortage of staff, lack of communication, defaulters, negative impact of the CCMDD programme and lack of training as challenges to the implementation of the CCMDD programme in Sekhukhune district clinics.

**Conclusion:**

Sekhukhune public clinics have challenges concerning the implementation of the CCMDD programme; therefore, it is very crucial to provide proper training about the programme to all PHC facility staff members to improve the CCMDD implementation.

**Contribution:**

The study would provide suggestions to implement the CCMDD programme by correcting the process of enrolling clients, the methods of packaging and dispensing the medications, sending SMSs, as well as delivery to the pick-up points.

## Introduction

Since 2016, several countries, especially the sub-Saharan Africa, have adopted and scaled up differentiated service delivery (DSD) as part of the national policy and for the adults established on antiretroviral therapy (ART) (World Health Organization [WHO] 2021). International AIDS Society (2024) defined DSD as a client-centred approach that simplifies and adapts human immunodeficiency virus (HIV) services across the cascade to reflect the preferences, expectations and needs of people living with and affected by HIV while reducing burdens on the health system. Differentiated service delivery was recommended by WHO in 2015, and evidence shows that it can enhance client outcomes including the quality of care, ensure that the health system functions efficiently and also enable the health system to refocus resources to the most in need (International AIDS Society 2024). South Africa has experienced an unpredicted growth in patients requiring access to long-term therapies over the past decades, which has placed strains on available resources and opportunities for external parties to support the care of chronic patients (National Department of Health 2022). According to Galal (2022), there were 4.74 million hypertension sufferers in 2019 in South Africa, which renders hypertension as the most occurring chronic condition of health in the country. Galal (2022) further indicated that diagnosed incidences in South Africa were nearly 1.6 million for HIV and acquired immunodeficiency syndrome (AIDS), diabetes with 1.677 million cases, arthritis with 1.218 million cases and asthma with 1.027 million cases. Public health facilities have become overcrowded because most of these chronic clients use free public service to receive their medications. The Central Chronic Medicines Dispensing and Distribution (CCMDD) programme was initiated in 2014 as a National Health Insurance (NHI) initiative to provide an alternative mechanism to facilitate access to medicines for stable patients (National Department of Health 2022). According to Health System Trust (HST 2020), CCMDD is a programme-driven distribution and dispensing of medicine from a central location for the benefit of patients whose conditions are chronic but are consistent on their medication. The CCMDD was operating in 16 districts at the start of March 2016 and was expanded to reach 46 districts from 8 participating provinces by January 2018 National Department of Health Annual Report 2020/2021 (2021). From March 2016 to October 2019, the number of health facilities increased from 972 to 3436, and the number of external pick-up points increased from 164 to 2037 and 4 321 755 patients enrolled to receive their medication through the CCMDD programme (The National Department of Health Annual Report 2020/2021 [2021]). Since Sekhukhune public clinics started implementing the CCMDD programme, the researcher observed that there is inconsistence in the delivery of CCMDD medications and that there is higher rate of antiretroviral (ARV) patient loss to follow-up and self-transfers, as well as mismanagement of clients, especially those with diabetes and HIV and AIDS. The aim of this study was to describe the challenges related to the implementation of the CCMDD programme in Sekhukhune district clinics. The Context, Input, Process, Product (CIPP) Evaluation Model was utilised because the researcher was looking for improvement of the CCMDD programme or its effectiveness by identifying the inherent potential and limitations in the implementation of the CCMDD programme.

### Aim

This study aims to describe the challenges related to the implementation of the CCMDD programme in Sekhukhune district clinics.

## Research methods and design

The qualitative research approach with an exploratory and descriptive research design was employed in this study. Such an approach enhanced the study’s intended goal of describing the sampled participants’ viewpoints and experiences concerning challenges in the implementation of the CCMDD programme in the selected Sekhukhune district clinics.

### Study setting

The study was conducted in nine Sekhukhune district primary health care (PHC) facilities. Sekhukhune is predominantly rural and is one of Limpopo province’s five district municipalities. The Sekhukhune District Municipality is situated in the southeastern part of Limpopo province. Furthermore, this municipality is made up of 4 local municipalities, 83 clinics, 4 community health centres (CHCs), 7 hospitals, 117 wards and a total of 764 villages.

The Sekhukhune district is populated by nearly 1.2 million people, most of whom are Bapedi.

### Study population and sampling strategy

Participants of this study comprised 27 nurses who are running the CCMDD programme in the Sekhukhune PHC facilities and 18 clients who have enrolled on the CCMDD programme for more than 6 months and utilised Sekhukhune PHC facilities as their pick-up points. In this study, the non-probability sampling approach was utilised on the strength that it enables a non-random sampling according to which the selection of a prospective participant cannot be guaranteed or known in advance of the actual start of the research (Polit & Beck 2020). Examples of non-probability sampling techniques are convenience sampling, purposive sampling, quota sampling and consecutive sampling (Polit & Beck 2020). In this study, the purposive sampling technique was utilised in the recruitment of participants, whereas the convenience sampling technique was used to select the nine Sekhukhune clinics.

### Data collection

Data were collected by the researcher using individual face-to-face (one-on-one) semi-structured interviews.

Two interview guides were prepared by the researcher and used to guide the conversion during the interview, one for nurses and one for CCMDD clients. Interviews were conducted in English for those who do not understand Sepedi and also in Sepedi for those who do not understand English. Each interview session lasted no more than 45 min duration. A digital recorder was utilised to capture the verbatim response of the interviewees, and field notes were utilised for both the participants’ non-verbal and verbal cues. After the interview sessions had ended, the researcher downloaded all digital copies and records to her password-protected personal computer to keep them secure and safe.

### Data analysis

Data were analysed thematically and transcribed by the researcher from the digital voice recorder and the field notes. The verbatim transcripts were sent to an independent coder to assist with data analysis. The coder was requested to sign a non-disclosure and confidential agreement with the researcher. Both the coder and the researcher discussed the coded data and reached a consensus concerning the problem being researched.

### Trustworthiness

To ensure reliability, the researcher ensured that all procedures to be followed during the study were transparent. Credibility was ensured through data triangulation – in this study, various data collection methods such as semi-structured interviews, field notes and digital voice recorder were utilised. Dependability was facilitated through an audit trail of the data analysis procedures and recorded data. Confirmability in this study was facilitated by means of member checking and peer debriefing to ensure that data accurately reflected the participant’s experiences. Transferability was ensured through the provision of a detailed description of the methodology of the study, participants and the context of the study. Authenticity was ensured through the use of direct quotes from participants to support each theme and subthemes.

### Ethical considerations

The study received ethical clearance from the University of South Africa’s College of Human Sciences with ethics clearance reference number: 60756349_CREC_CHS_2023. Permission to conduct the study was obtained from the Sekhukhune district PHC office, the provincial office and local area managers of the nine selected PHC facilities in the Sekhukhune District Municipality by the Department of Health Limpopo ethics committee with permission reference number: LP_2023-10-002. All participants provided informed consent after a detailed description of the scope of the study goals. The interviews were held in a pre-requested private room, and codes or pseudonyms such as Operational manager (OPM), Assistant operational manager (AOPM), Professional nurse (PN), Professional nurse with speciality (PNS), Enrolled nurse (EN), Enrolled nursing auxiliary (ENA), Client (C) and Participant (P) were used instead of using their real names. Participants were assured that their personal information, and all study-related data would be treated in strict confidence.

## Findings

### Characteristics of the study participants

Forty-five participants were interviewed from nine clinics as each clinic had five participants. Twenty-seven nurses, aged 30–58 years, and 18 CCMDD clients, aged 30–70 years, formed the sample for this study. Table 1 and Table 2 show the characteristics of the nurses and the CCMDD clients, respectively.

**TABLE 1:** Study sites and characteristics of nurses.

Participant number	Name of the clinic	Age (years)	Gender	Years of experience	Level of qualification	Category
1	Clinic B	46	Female	17	Diploma	Professional nurse with PHC speciality
2	Clinic B	52	Female	12	Diploma	Professional nurse with PHC speciality
3	Clinic C	54	Female	29	Honours degree	Operational manager
4	Clinic C	39	Female	10	Certificate	Enrolled nursing auxiliary
7	Clinic B	41	Male	15	Diploma	Enrolled nurse
10	Clinic D	35–49	Female	10 +	Certificate	Enrolled nurse
12	Clinic D	34	Female	8	Bachelor’s degree	Professional nurse with PHC speciality
13	Clinic A	45	Female	10	Diploma	Professional nurse
14	Clinic A	52	Female	24	Bachelor’s degree	Operational manager
16	Clinic A	52	Female	22	Certificate	Enrolled nurse
17	Clinic E	50	Female	16	Bachelor’s degree	Acting Operational manager
20	Clinic E	42	Female	10	Bachelor’s degree	Professional nurse
21	Clinic F	43	Female	16	Bachelor’s degree	Acting Operational manager
22	Clinic F	51	Female	12	Certificate	Enrolled nursing auxiliary
23	Clinic F	58	Female	33	Diploma	Professional nurse
26	Clinic G	55	Male	34	Bachelor’s degree	Operational manager
27	Clinic G	46	Female	17	Diploma	Enrolled nurse
29	Clinic H	58	Female	25	Diploma	Professional nurse with PHC speciality
28	Clinic H	40	Female	18	Certificate	Enrolled Nurse
34	Clinic I	50	Female	19	Bachelor’s degree	Professional nurse with PHC speciality
33	Clinic E	38	Female	12	Certificate	Enrolled nursing auxiliary
35	Clinic I	49	Female	25	Certificate	Enrolled nurse
36	Clinic D	42	Female	15	Diploma	Acting Operational manager
37	Clinic I	34	Female	8	Diploma	Professional nurse
38	Clinic C	38	Female	14	Diploma	Professional nurse
39	Clinic G	30	Female	8	Diploma	Professional nurse
40	Clinic H	50	Female	5	Diploma	Professional nurse

PHC, primary health care.

**TABLE 2:** Study sites and characteristics of the central chronic medicines dispensing and distribution clients.

Clinic name	Participant number	Age (years)	Gender	Level of qualification	CCMDD status	Name of chronic illness
A	15	42	Female	Passed Grade 12	Enrolled	HIV and AIDS
	30	38	Female	Failed Grade 12	Enrolled	HIV and AIDS
B	8	56	Female	Standard five dropout	Enrolled	HIV and AIDS, HPT
	9	38	Female	Certificate	Enrolled	HIV and AIDS
C	4	74	Female	None	Enrolled	HPT
	5	55	Female	Passed Grade 12	Enrolled	Migraine
D	10	37	Male	Passed Grade 12	Enrolled	HIV and AIDS
	31	30	Female	Certificate	Enrolled	HIV and AIDS
E	18	65	Male	Passed standard 3	Enrolled	Asthma
	19	74	Female	Standard 3 dropout	Enrolled	HPT
F	24	68	Female	None	Enrolled	HPT
	25	45	Female	Passed Grade 11	Enrolled	HIV and AIDS
G	44	53	Female	Grade 11 dropout	Enrolled	HIV and AIDS
	45	47	Female	Passed Grade 12	Enrolled	HIV and AIDS
H	32	45	Female	Grade 6 dropout	Enrolled	HIV and AIDS
	43	44	Female	Certificate	Enrolled	HIV and AIDS
I	41	45	Female	Certificate	Enrolled	HIV and AIDS
	42	34	Female	Failed Grade 12	Enrolled	HIV and AIDS

CCMDD, central chronic medicines dispensing and distribution; HIV, human immunodeficiency virus; AIDS, acquired immunodeficiency syndrome; HPT, hypertension.

In this study, the quotes from participants were written in the manner in which they present the category of the participant, followed by the participant number and lastly the clinic name.

### Themes

One theme and five subthemes were formed during data analysis, and Table 3 presents the theme and subthemes:

**TABLE 3:** Theme and subthemes.

Theme	Subthemes
1. Challenges related to the implementation of the CCMDD programme	1.1 Staff shortage 1.2 Lack of communication 1.3 Defaulters 1.4 Negative impact of the CCMDD programme 1.5 Lack of training

CCMDD, central chronic medicines dispensing and distribution.

### Theme: Challenges related to the implementation of the central chronic medicines dispensing and distribution programme

Participants of the study revealed that they have encountered several challenges as the CCMDD was implemented in their facility. Shortage of staff, lack of communication, defaulter, negative impact of the CCMDD and lack of training emerged as subthemes when the participants of the study were sharing their challenges.

#### Staff shortage

Participants of the study expressed how the shortage of staff impacts the implementation of the programme in their facility. The quotes of one participant are outlined below:

‘Sometimes clients come for review in large numbers while there is shortage of staff in our facility, so you have to deal with a lot of CCMDD paper work and other clients who came for other services, as a result conflict arise between the patients and nurses because queues are no longer moving as expected.’ (PNSP2B)

‘There is a shortage of staff. There must be a focal person for the CCMDD programme who will be responsible for sorting out patients, enroll clients on CCMDD programme, giving them health education, tracing their medication, and supplying them with their medication parcel.’ (PNP13A)

‘Time of enrollment is long, so clients become impatient with us whereas we are short staffed. As a result, it creates conflict between the nurses and the patients.’ (OPMP14A)

The participants of the study acknowledge that the programme takes a lot of time, and it needs enough staff to run while most health facilities are short staffed. Central chronic medicines dispensing and distribution programme requires staff such as nurses, doctors and pharmacist to capture data, as well as a focal person who will be responsible for facilitating the implementation of the programme in the facility. For a PHC facility to be fully functional, enough staff is needed. Nursing shortage can lead to errors, higher morbidity and mortality rates (Haddad, Annamaraju & Toney-Buttler 2023).

#### Lack of communication

Effective communication at work helps to reduce errors that result in the guarantee of patient safety; and it also reduces workplace conflicts, improving synergy and productivity (Indeed Editorial Team 2022). Participants of the study indicated that there are no proper channels of communication between the pharmacy direct and the facility, which led to the poor implementation of the CCMDD programme and created conflict between nurses and clients. These poor channels of communication are characterised by late, wrong and no deliveries of medications, no collection of scripts and no short message service (SMS) notification. One participant’s response is outlined below:

‘There is late collection of forms from the facility, whereby we fill up forms but no one comes to collect them and when they do come they will tell you that the forms have been expire, as a result the moral of the staff become demoralised and they become reluctant to fill up forms. And when forms are no longer filled up, the facility becomes congested again. Medications do not come on time or sometimes not being delivered at all and it also demoralises the staff because to re-fill the forms again and even client’s loss trust on us.’ (OPMP14A)

‘Patients no longer receiving SMS notifications some do not come to the clinic because they were not reminded by the SMS that they were supposed to get. They just come after few days to check if they do have delivery or not.’ (PNP20E)

‘Client gets SMS but when she or he arrives in the facility, you find that the medication is not there. Sometimes we receive the medications that do not belong to our clients nor clinic, different names that we do event even recognise.’ (ENP10D)

Central chronic medicines dispensing and distribution clients also have revealed that they have experienced challenges in terms of communication and their responses are quoted below:

‘Sometimes, we do not get the SMS notification but the medication is already being delivered in the facility.’ (CP15A)

‘The challenge is that, sometimes you do not get you medications, so you have to queue with other patients which results in waste of time and money.’ (CP30A)

‘My medication has been delivered to another clinic not the one that has enrolled me and now people that I work with know my status.’ (CP44G)

Participants of the study emphasised that lack of communication has led to problems such as late and non-delivery of medications, inconsistency in sending SMS notifications, late collection and non-collection of medication scripts from the facilities, wrong deliveries of medications, wrong contact details, mismanagement of clients, especially HIV and AIDS clients, and incomplete medication packages.

#### Defaulters

Participants of the study revealed treatment defaulters as one of the challenges that they encounter when running the CCMDD programme. Their responses are outlined below:

‘CCMDD clients do not come to the clinic on time to collect their medication. Even if they receive some SMS notification, they will just sit at home and come some other time. We are not sure if they are taking their medications correctly because for example HIV clients when you tell them that their virus is suppressed and that they must be enrolled on the CCMDD programme, it seems as we are cursing them because most of HIV CCMDD enrolled client’s their viral load becomes high when we collects their due bloods and we are not sure if they become more relaxed or not and no longer adhering on treatment when they are at home. So, we end up failing to renew their scripts because of high viral load.’ (PNP13A)

‘Clients do not come to collect their medications when they are delivered, and we have a bunch of uncollected CCMDD medications in our facility and we ask ourselves that what are these people drinking at home because their medications are with us?’ (AOPMP21F)

‘Client usually defaults or sometimes they sent people to collect their while they know that it’s time to collect their blood samples.’ (PNP40H)

The study results emphasised that the CCMDD programme has increased the rate of defaulters because most of the clients enrolled on the CCMDD programme do not honour their appointment dates. As a result, facilities have a bunch of uncollected medications, and blood samples that are due for collection are also missed to be collected in time or not collected at all.

#### Negative impact of the central chronic medicines dispensing and distribution programme

The participants of the study revealed that the CCMDD programme has a negative impact on the facility and clients, which leads to poor implementation of the programme in their facility. Their response is supported by the following quotes:

‘CCMDD Programme reduces our clinic statistic and government will not hire new staff for us because they will tell you that your statistic is low, so the staff that you have is enough.’ (PNSP1B)

‘CCMDD programme is more interested on reducing overcrowding of clients in the facility rather than patient care because most of the clients who are enrolled on the CCMDD programme they enjoy staying at home and sending people to collect medication on their behalf even if they become ill they do not come to the clinic until their conditions complicate, as a results some develop gangrene and have their legs being amputated.’ (PNSP1B)

‘CCMDD has created more deaths among our chronic clients than improving life. This is because when they are ill, they do not come to the clinic; they enjoy being away from us or the clinic. They will just stay at home until their condition becomes worse.’ (PNSP2B)

‘There is overloading of clients with medication because clients receive SMS informing him or her that his or her medication has been delivered but when he/comes to the clinic you find that the medication is not yet been delivered, so we give the client clinic medication stock rather than returning him or her home empty handed.’ (PNSP2B)

‘We have learnt that our patients especially those with HIV are enrolled on the programme with suppressed viral load but when we it is time to review their bloods, most of them their viral loads become higher ad we ask ourselves why there is increase in viral load while they are taking their medications. We are not sure what is going on with HIV CCMDD enrolled clients.’ (ENP16A)

‘Clients on CCMDD programme miss opportunities to be given health education.’ (PNSP2B)

The study findings shows that he CCMDD programme has a negative impact that is characterised by a reduction in clinic statistics, negligence of patients, creation of more deaths than improving life and also patients missing opportunities to be given health education.

#### Lack of training

The participants of the study pointed out that they need formal training about the programme as some of them use the internet to guide them when implementing the programme. Responses of the participants are quoted below:

‘Every sister who is running the CCMDD programme must have proper in- service training because some of us have never attended any CCMDD training and we are unsure of what is needed and what is not needed.’ (PNSP2B)

‘Some of nurses do not understand the criteria of enrollment, which is why most of the time we miss clients who qualify to be on the programme.’ (PNSP34I)

‘Sometimes you find that the forms of the patient were not filled correctly when he or she was enrolled which led to many errors, for example wrong medication.’ (ENAP6C)

Proper in-service training is necessary for a programme like CCMDD to be implemented smoothly. Participants of the study acknowledged that some of the nurses do not understand the CCMDD criteria for enrolment and that’s why mistakes during enrolment occurred, and they emphasised the importance of proper training on the CCMDD programme in order to correct such mistakes.

## Discussion

The study findings presented a full picture of the challenges that nurses and CCMDD clients encounter with the implementation of the CCMDD programme. This study revealed that the CCMDD programme in the Sekhukhune district has challenges such as staff shortage, lack of communication, defaulters, negative impact of the CCMDD programme and lack of training, and these challenges have a negative impact on the implementation of the CCMDD programme in the Sekhukhune PHC facilities. The participants of the study revealed that there is a shortage of staff and a focal person for the programme, whereas sometimes clients come in large numbers for review and enrolment. The findings are supported by Muthelo et al. (2020) in their study; they revealed that PHC facilities are faced with the challenge of understaffing as the available staff also did not receive formal training on the implementation of the CCMDD programme. Similar findings are also described by Belay et al. (2022) who revealed that challenges to DSD model implementation include staff shortages, lack of information on the model implementation and lack of staff clarity on eligibility criteria, and all these challenges have policy implications to avail sufficient numbers and a diverse range of DSD workers who are given the necessary training, skills and tools to ensure that the model is implemented with competence, responsiveness and productivity. Bogart et al. (2022) in their study emphasised that there should be dedicated CCMDD staff with specific tasks such as providing refills, pulling files and tracking patients who missed refill pick-up points in order to implement the programme effectively. The study found that CCMDD clients do not honour their appointment dates and sometimes they even default for some weeks. Bogart et al. (2022) also supported these study findings because their study revealed that some patients are not loyal to their dates; they usually come 2 days or after a week to collect their medication. Participants of the study conducted by Monyela (2021) also indicated that some patients miss their appointments and delay to go to the pick-up points, and when they do come, they find that their medication parcels are no longer there because they are returned to the depot 7 days after the collection date. Sekhukhune PHC facilities experience communication challenges such as late collection of scripts that end up expired while still in the facility and results in re-enrolment of clients, non-collection of scripts, non-delivery of medication, wrong deliveries of medication parcels, delayed deliveries of medication parcels while clients have already been sent a SMS, incomplete medication parcel patients no longer getting SMS notifications, failure to collect viral load bloods for HIV and AIDS clients that are due, provision of wrong personal information and contacts by patients, and these challenges were described by the participant of the study. Participants of the study conducted by Muthelo et al. (2020) also noted that sometimes they receive collecting messages at the facility but when they arrive, they find that their medication is not yet being delivered. Tariq et al. (2022) in their study revealed that medication errors can occur at many steps in patient care, from ordering or prescribing, documenting, transcribing, dispensing, administering and monitoring. Bogart et al. (2022) also noted that patients had communications issues with the distributor regarding the text reminders for refills whereby text reminders were not delivered, specified incorrect date or suggested that the refill was available at the pick-up point before it was ready. Bogart et al. (2022) also reported that there is poor communication between clinics and pick-up point provider, which led to confusion around whether patients had defaulted or collected their medication at the clinic rather than the external pick-up points and around the understanding of why the patient’s medication was not available at the external pick-up point. The findings of the study revealed that the CCMDD programme has a negative impact on the facility that ranges from reducing clinic statistic and focusing on the reduction of overcrowding rather than quality patient care; it has created more deaths among chronic clients than improving life. This study is supported by the participants of the study conducted by Dorwad et al. (2020), who revealed that there is no time for health care workers to equip patients with competencies required to engage with HIV services and learn about taking their ART as a result of rushed clinical appointments. The study also raised a concern that CCMDD could delay clients from seeking medical attention if they became ill (Dorward et al. 2020). The study findings also revealed a lack of training as a challenge that affects the implementation of the CCMDD programme. The findings are supported by Bogart et al. (2022) in their study titled ‘Implementation of South Africa’s Central Chronic Medicine Dispensing and Distribution Program for HIV Treatment’, which reported that inadequately trained staff led to errors in prescriptions, in filing patient records, in tracking patients, in enrolling ineligible patients or not enrolling eligible patients.

## Conclusions

The purpose of this study was to describe the challenges related to the implementation of the CCMDD programme in Sekhukhune district clinics. Sekhukhune public clinics have challenges with regard to the implementation of the CCMDD, and these challenges emanated from the shortage of staff, lack of communication, defaulters, negative impact of the CCMDD programme and lack of training. Therefore, it is very crucial to provide proper training about the programme to all Sekhukhune PHC facility staff members in order to improve the CCMDD implementation. Future research should focus on investigating on non-delivery of CCMDD medications to the chosen external points.

### Limitations

The results of this study cannot be generalised because it was conducted in two Sekhukhune sub-districts, Fetakgomo Tubatse and Makhuduthamaga.
